# Risk factors for SARS-CoV-2 seroprevalence following the first pandemic wave in UK healthcare workers in a large NHS Foundation Trust

**DOI:** 10.12688/wellcomeopenres.17143.1

**Published:** 2021-09-03

**Authors:** Hayley Colton, David Hodgson, Hailey Hornsby, Rebecca Brown, Joanne Mckenzie, Kirsty L. Bradley, Cameron James, Benjamin B. Lindsey, Sarah Birch, Louise Marsh, Steven Wood, Martin Bayley, Gary Dickson, David C. James, Martin J. Nicklin, Jon R. Sayers, Domen Zafred, Sarah L. Rowland-Jones, Goura Kudesia, Adam Kucharski, CMMID COVID-19 Working Group, Thomas C. Darton, Thushan I. de Silva, Paul J. Collini

**Affiliations:** 1 South Yorkshire Regional Department of Infection and Tropical Medicine, Sheffield Teaching Hospitals Nhs Foundation Trust, Sheffield, S10 2JF, UK; 2 Department of Infection, Immunity and Cardiovascular Disease, University of Sheffield, Sheffield, S10 2TN, UK; 3 Centre for Mathematical Modelling of Infectious Diseases, London School of Hygiene and Tropical Medicine, London, WC1E 7HT, UK; 4 Academic Directorate of Communicable Diseases and Specialised Medicine, Sheffield Teaching Hospitals Nhs Foundation Trust, Sheffield, S10 2JF, UK; 5 Department of Scientific Computing and Informatics, Sheffield Teaching Hospitals Nhs Foundation Trust, Sheffield, S10 2JF, UK; 6 Department of Chemical and Biological Engineering, University of Sheffield, Sheffield, S10 2TN, UK; 7 The Florey Institute for Host-Pathogen Interactions, University of Sheffield, Sheffield, S10 2TN, UK; 8 Sheffield Institute for Nucleic Acids, University of Sheffield, Sheffield, S10 2TN, UK; 9 Department of Virology, Sheffield Teaching Hospitals Nhs Foundation Trust, Sheffield, S5 7AU, UK

**Keywords:** Seroprevalence; antibody; Healthcare Worker; SARS-CoV-2; COVID; modelling; age; risk

## Abstract

**Background:** We aimed to measure SARS-CoV-2 seroprevalence in a cohort of healthcare workers (HCWs) during the first UK wave of the COVID-19 pandemic, explore risk factors associated with infection, and investigate the impact of antibody titres on assay sensitivity.

**Methods:** HCWs at Sheffield Teaching Hospitals NHS Foundation Trust were prospectively enrolled and sampled at two time points. SARS-CoV-2 antibodies were tested using an in-house assay for IgG and IgA reactivity against Spike and Nucleoprotein (sensitivity 99·47%, specificity 99·56%). Data were analysed using three statistical models: a seroprevalence model, an antibody kinetics model, and a heterogeneous sensitivity model.

**Results:** As of 12th June 2020, 24·4% (n=311/1275) of HCWs were seropositive. Of these, 39·2% (n=122/311) were asymptomatic. The highest adjusted seroprevalence was measured in HCWs on the Acute Medical Unit (41·1%, 95% CrI 30·0–52·9) and in Physiotherapists and Occupational Therapists (39·2%, 95% CrI 24·4–56·5). Older age groups showed overall higher median antibody titres. Further modelling suggests that, for a serological assay with an overall sensitivity of 80%, antibody titres may be markedly affected by differences in age, with sensitivity estimates of 89% in those over 60 years but 61% in those ≤30 years.

**Conclusions: ** HCWs in acute medical units working closely with COVID-19 patients were at highest risk of infection, though whether these are infections acquired from patients or other staff is unknown. Current serological assays may underestimate seroprevalence in younger age groups if validated using sera from older and/or more symptomatic individuals.

## Introduction

Healthcare workers (HCWs) are at increased risk of COVID-19 ^1, 2^. The true number of HCWs exposed to SARS-CoV-2 to-date is unknown, particularly during the early stages of the pandemic. Initial methods to estimate HCW exposure included extrapolation from work absenteeism rates, and are unlikely to be reliable ^3^. Confirmation by molecular testing increased the accuracy of case detection, although access to nucleic acid amplification testing (NAAT) was limited during the early stages of the pandemic in the UK ^4^. Detection of exposure by antibody seroconversion may provide a more accurate estimate of risk in HCW populations, can be performed at large scale, and is less affected by symptom-activated testing pathways ^5– 8^.

To enable the accurate interpretation of seroprevalence readouts, detailed characterisation of antibody evolution relative to the sampling time-frame, immunoglobulin isotype, antigenic target and assay performance is required ^9– 14^. Many antibody assays have been evaluated using samples from hospitalised patients; it is unclear how these assays perform with the lower antibody levels found in those with more mild or asymptomatic SARS-CoV-2 infection ^10, 12^. Furthermore, while antibody levels to some coronaviruses are higher in older individuals, it is unclear whether this results from a higher risk or exposure to the virus, greater antigenic load or boosting of antibodies from previous seasonal coronavirus infections ^15– 19^. This may also lead to age-specific differences in antibody assay sensitivity, which could be a significant confounder in population seroprevalence studies.

In this study we measure SARS-CoV-2 seroprevalence in HCWs at Sheffield Teaching Hospitals NHS Foundation Trust (STH), following the first wave of the pandemic in the UK. We model the kinetics of the evolving antibody response, the impact of age on assay sensitivity and explore risk factors associated with infection.

## Methods

### Background and setting

STH offers secondary- and tertiary-level care across four sites in South Yorkshire, UK, and has 1,669 inpatient beds and 18,500 employees ^20^. The first patient with confirmed COVID-19 was admitted to STH on 23 February 2020; the first wave of the UK pandemic occurred between March 2020 and June 2020.

Testing of symptomatic staff for SARS-CoV-2 by NAAT was introduced on 17 March 2020. On the same day, Public Health England (PHE) de-escalated the recommendations for the personal protective equipment (PPE) required by HCWs caring for inpatients with suspected or confirmed COVID-19 from ‘Level 3 Airborne’ to ‘Level 2 Droplet’ for routine care ^21^. Subsequently, the requirement for universal ‘Level 2 Droplet’ PPE for all inpatient and outpatient care began on 08 April 2020. Local STH policy was changed on 15 June 2020 to mandate staff use surgical face masks while on hospital premises.

### Recruitment and consent

From 13–18 May 2020, all contactable STH staff (n=17,757) were invited to take part in the COVID-19 Humoral ImmunE RespOnses in front-line HCWs (HERO) study by email and intranet alert. To engage staff in areas with limited communications access, additional recruitment posters and face-to-face enrolment sessions were used.

Following an electronic informed consent process, participants provided self-reported data on-line on age, gender, ethnicity, job role, and pandemic working environment (‘COVID-19 zones’) ^21^. Details of any possible or confirmed prior COVID-19 illnesses occurring since 01 February 2020 was also collected. These were categorised as: i), diagnosed with COVID-19 and confirmed by NAAT, ii), clinically diagnosed with COVID-19 but NAAT not performed, and iii), self-reported symptoms only ^21^. Together, we defined these three groups as “symptomatic”, as asymptomatic testing was only introduced after the study recruitment period. Those reporting no illness between 01 February 2020 to the date of recruitment were defined as “asymptomatic”. All that had enrolled were emailed times of phlebotomy appointments, and were invited to attend on a first come first served basis for the first visit, and then invited by email to book a specific appointment slot to attend for their second visit after four weeks +/- 7 days. An 8.5ml serum sample was taken at each visit to outpatient phlebotomy services for serological testing.

### SARS-CoV-2 serology

Serum samples were tested for IgG and IgA reactivity to two SARS-CoV-2 proteins using an in-house ELISA: the full-length extracellular domain (amino acids 14-1213) of Spike glycoprotein, including a replacement of the furin cleavage site R684-R689 by a single alanine residue and replacement of K986-V987 by PP, produced in mammalian cells; and full-length untagged Nucleocapsid protein (NCP) produced in *E. coli* ( Uniprot ID P0DTC9 (NCAP_SARS2)) *.* ^22– 24^. High binding microtitre plates (Immulon 4HBX; Thermo Scientific, 6405) were coated overnight with proteins diluted in phosphate buffered saline, washed with 0·05% PBS-Tween, and blocked for one hour with 200 µL/well casein buffer. Following optimisation, sample dilutions used were 1:200 for the IgG assay or 1:100 for the IgA assay ^21^. Plates were emptied and 100 µL/well of sample or control loaded. After two hours incubation, plates were washed and loaded with goat anti-human IgG-HRP conjugate (Invitrogen, 62-8420) at 1:500, or goat anti-human IgA-HRP conjugate (Invitrogen, 11594230) at 1:1000, for one hour. Plates were washed and developed for 10 minutes with 100 µL/well TMB substrate (KPL, 5120-0074). Development was stopped with 100 µL/well HCl Stop solution (KPL, 5150-0021), and absorbance read at 450nm. All steps were performed at room temperature.

A calibration curve of sera pooled from convalescent SARS-CoV-2 NAAT-confirmed patients with high antibody titres for both spike and NCP was included on plates to allow quantification of antibody concentrations. The calibration curve was generated by serially diluting in 1·75× steps from a starting concentration of 1:200 for the IgG assay or 1:100 for the serum IgA assay. When the WHO International Standard for anti-SARS-CoV-2 immunoglobulin (NIBSC, 20/136) later became available, the calibration curve was run in parallel for the IgG assay ^21^. Data for the IgG assay are therefore given in WHO antibody units, whereas IgA assay data are given in arbitrary antibody units.

### Sample size

To meet the primary objective of measuring the SARS-CoV-2 IgG seroprevalence, we calculated a sample size of 1,000 HCWs would provide +/-1·4% precision based on a seroprevalence estimate that ~4% of the UK population may have been infected by April 2020, with a two-sided 95% confidence interval (with n=753, Binomial exact 95%CI has been estimated to be 2·7-5·6%) ^25^.

### Statistical modelling

We considered three statistical models, i) a seroprevalence model, ii) an antibody kinetics model, and iii) a heterogeneous sensitivity model. For the seroprevalence model, we used the serostatus of all participants at first blood draw in a sensitivity- and specificity-adjusted Bayesian multilevel logistic regression model. Using seropositivity as the binary response variable, we considered three different model subtypes with varying primary exposures; job location, contact with COVID-19 patients, and job type ^21^. In addition, we fitted a symptomatic prevalence model, where the data used were seropositive persons only, and the binary response variable was asymptomatic or symptomatic infection.

For the antibody kinetics model, we included samples from seropositive individuals in a Bayesian multilevel linear regression model in two parts: i) using log2 antibody units (logAU) at the first blood draw as the response variable and ii) using the change in antibody titre at the follow up bleed (median 28 days) as the response variable. Age, ethnicity, gender and symptom severity (asymptomatic or symptomatic) were used as covariates and each model was run separately for four different antibody-antigen combinations; Spike-IgG, NCP-IgG, Spike-IgA, NCP-IgA. The time until seroreversion was calculated for each covariate group and antibody-antigen interactions by i) sampling a starting titre value and a rate of decline from the two models, and then ii) calculating the time until the minimum observed antibody value was reached for that antibody-antigen interaction, assuming a continuous rate of decrease.

In our heterogeneous sensitivity and specificity model, we explored how estimates derived from our assay validation dataset generalise to covariate groups, e.g. participant age. To model the generalisability of these performance measures, we compared the seropositivity classification of our study dataset using our in-house antibody assay, with the predicted seropositivity classification from hypothetical assays with a quoted sensitivity and specificity. Our model considers the different distribution of the *A*_450_ values in the assay validation and HERO study datasets to model how reliably quoted performance measures generalise. Using the assay validation dataset, we estimated the *A*_450_ cut-off value for a range of chosen sensitivity values, and then used this *A*_450_ cut-off to classify seropositivity in the study dataset. We then estimated the implied sensitivity on the HERO dataset by comparing seropositivity classification based on the estimated *A*_450_ cut-off value, with the seropositivity classification from our in-house assay (which for ease of comparison, we assume represents the maximum possible sensitivity and specificity (i.e. 100%) in this model). This framework allowed us to estimate the hypothetical performance of serological assays reported in the literature on our HERO dataset, along with co-variate specific sensitivity.

All analysis was performed in R version 4.0.2 and cmdstanr version 0.2.0. An R package containing all the analysis in this study is available at https://doi.org/10.5281/zenodo.5215671.

### Regulatory review

Following internal scientific review, local R&D (5 May 2020 ref: STH21394) and HRA and Health and Care Research Wales (HCRW) approval were given (29 April 2020 ref: 20/HRA/2180, IRAS ID: 283461). Anonymised serum samples from hospitalised COVID-19 patients and serum collected prior to 2017 during routine clinical care were used for assay validation with approval from STH R&D office.

## Results

### Serology assay development

The assay validation dataset ^26^. consisted of serum from 190 SARS-CoV-2 NAAT-confirmed cases (52 hospitalised patients and 138 healthcare workers with mild infections sampled between 14 and 120 days from NAAT positivity), and 675 patients sampled prior to 2017 ( *Extended data:* Table S1). Thresholds for defining reactivity to spike ( *A*_450_ 0·1750) or NCP ( *A*_450_ 0·1905) were set to optimise the sensitivity of each assay. Given the IDSA guidance for ensuring a specificity of ≥99·5% in assays used for SARS-CoV-2 seroprevalence studies, specificity was enhanced by defining a SARS-CoV-2 seropositive sample as one where both spike and NCP were reactive ^27^. This resulted in a sensitivity of 99·47% (95% confidence interval (CI) 97·10% - 99·99%) and specificity of 99·56% (95% CI 98·71% - 99·91%) for our IgG assay ( *Extended data:* Figure S1). Rapid waning of IgA responses following SARS-CoV-2 infection complicated defining positive and negative samples based on the convalescent sera we used for assay validation. We also observed more background reactivity for IgA compared to IgG in pre-pandemic samples. We therefore opted to use our spike and NCP IgA ELISA purely for comparing IgA levels in individuals classified as seropositive in our IgG assay ( *Extended data:* Figure S2). Antibody units at each given dilution of the calibration curve are shown in Table S2 ( *Extended data)*.

### Registration and study visits

1478 STH staff consented to take part between 13 May and 5 June 2020 ( *Extended data:* Figure S3). Of these, 1277 attended for a first visit (V1) between 15 May 2020 and 12 June 2020. As two samples were contaminated in transit, we obtained a valid serostatus for 1275 samples. 1174 attended for a second visit (V2) between 15 June and 10 July 2020 ( *Extended data:* Figures S3 and S4).

### Demographics, job role, work locations, and environment

The majority of participants were female (n=1008/1275, 79·1%) and most described their ethnicity as white (n=1130/1275, 88·6%, Table 1). Nurses (433/1275, 33·9%), doctors (232/1275, 18·2%), health care assistants (163/1275, 12·8%), and domestic services staff (136/1275, 10·7%) constituted the largest proportion of job roles. Almost half (593/1275, 46·5%) of HCWs worked in parts of the hospital managing acute COVID-19 admissions including the Emergency Department (ED), Acute Medical Unit (AMU), Critical Care, and inpatient medical wards (respiratory, geriatric care, infectious diseases). Participants reported working in areas with the highest level of COVID-19 patient contact (red zones), either most days (n=423/1275, 33·2%) or occasionally (n=305/1275, 23·9%).

**Table 1.** Characteristics and serostatus of recruited participants who had a valid baseline result.

	Recruited with an initial valid serostatus	Seropositive at V1 (%)	Asymptomatic (% of seropositive at V1)	Completed both V1 and V2 (% of recruited)	Seroincident cases (% of seronegative at V1)
Total	1275	311 (24·4)	122 (39·2)	1166 (87·5)	16 (1·7%)
Job location					
Emergency Department (ED)	103	26 (25·2)	13 (50·0)	90 (87·4)	1 (1·2)
Acute Medical Unit (AMU)	83	38 (45·8)	17 (44·4)	66 (79·5)	0 (0·0)
Critical Care	100	18 (18·0)	7 (38·9)	95 (95·0)	0 (0·0)
Geriatric Care	23	3 (13·0)	1 (33·3)	22 (95·7)	1 (5·0)
Infectious Disease Ward	139	26 (18·7)	11 (42·3)	121 (87·1)	7 (6·2)
Other	664	157 (23·6)	56 (35·7)	621 (93·5)	2 (0·3)
Respiratory Geriatric Ward	92	27 (29·3)	10 (37·0)	85 (92·4)	2 (3·1)
Respiratory Ward	58	13 (22·4)	5 (38·5)	54 (93·1)	2 (4·4)
Job role					
Admin	127	26 (20·5)	12 (46·2)	118 (92·9)	1 (0·9)
Allied medical ^1^	38	0 (0·0)	—	37 (97·4)	0 (0·0)
Domestic services	136	39 (28·7)	24 (61·5)	127 (93·4)	4 (4·1)
Healthcare assistants	163	39 (23·9)	21 (53·8)	140 (85·9)	3 (2·4)
Doctors	232	52 (22·4)	18 (34·6)	211 (90·9)	0 (0·0)
Nurses	433	116 (26·7)	34 (29·3)	391 (90·3)	7 (2·2)
Other	31	5 (16·1)	2 (40·0)	29 (93·5)	0 (0·0)
Pharmacists	35	8 (22·8)	5 (62·5)	33 (94·3)	1 (3·7)
Occupational and physiotherapists	33	15 (45·5)	3 (20·0)	33 (100·0)	0 (0·0)
Radiographers	42	9 (21·4)	2 (22·2)	42 (100·0)	0 (0·0)
COVID-19 zone ^2^					
1 (lowest COVID-19 contact)	104	22 (21·2)	10 (54·5)	96 (92·3)	1 (1·2)
2	248	50 (20·2)	27 (46·0)	232 (93·5)	0 (0·0)
3	41	7 (17·1)	6 (14·3)	39 (95·1)	1 (2·9)
4	153	35 (22·9)	24 (31·4)	142 (92·8)	1 (0·8)
5	305	69 (22·6)	46 (33·3)	280 (91·8)	3 (1·3)
6 (highest COVID-19 contact)	423	128 (30·3)	76 (40·6)	376 (88·9)	10 (3·4)
Age (years)					
<30	267	67 (25·1)	32 (47·8)	236 (88·4)	6 (3·0)
30–39	306	69 (22·5)	22 (31·8)	279 (91·2)	5 (2·1)
40–49	314	72 (22·9)	29 (40·2)	293 (93·3)	2 (0·8)
50–59	314	76 (24·2)	28 (36·8)	288 (91·7)	1 (0·4)
60+	74	27 (36·5)	11 (40·7)	70 (94·6)	2 (4·3)
Ethnicity					
White	1130	281 (24·9)	108 (38·4)	1035 (91·6)	15 (1·8)
Black/Black British	33	6 (18·2)	3 (50·0)	30 (90·9)	0 (0·0)
Asian/Asian British	76	17 (22·4)	7 (41·2)	70 (92·1)	1 (1·7)
Other	33	7 (21·2)	4 (57·1)	30 (90·9)	0 (0·0)
Gender ^3^					
Female	1008	253 (24·1)	105 (41·5)	922 (91·5)	14 (1·9)
Male	265	58 (21·9)	17 (29·3)	242 (91·3)	2 (0·9)

^1^Allied Medical includes Speech and Language Therapists, Cardiac Physiologists, Dental Hygienists, Dietitians, ECG technicians, Orthotists, Podiatrists, Rehabilitation assistants ^2^COVID-19 Zones are defined in extended data ^21^. ^3^Participants were able to define their gender as non-binary, transgender or could choose not to disclose

### Unadjusted seroprevalence

Analysis of V1 samples revealed that 24·4% (n=311/1275) of HCWs were seropositive by 12 June 2020 ( Table 1). Of these, 39·2% (n=122/311) did not report a prior illness consistent with COVID-19. The second blood draw occurred a median of 28 days following the first visit (IQR 27-31) and 1166 had a valid V2 serology result ( *Extended data:* Figure S3). Comparison of serology data from both visits demonstrated that 16 out of 964 participants had seroconverted and 9 out of 311 participants seroreverted (i.e. loss of reactivity against either spike ( *A*_450 _<0·175) or NCP ( *A*_450_ <0·1905)).

### Adjusted seroprevalence estimates

**Figure 1.**
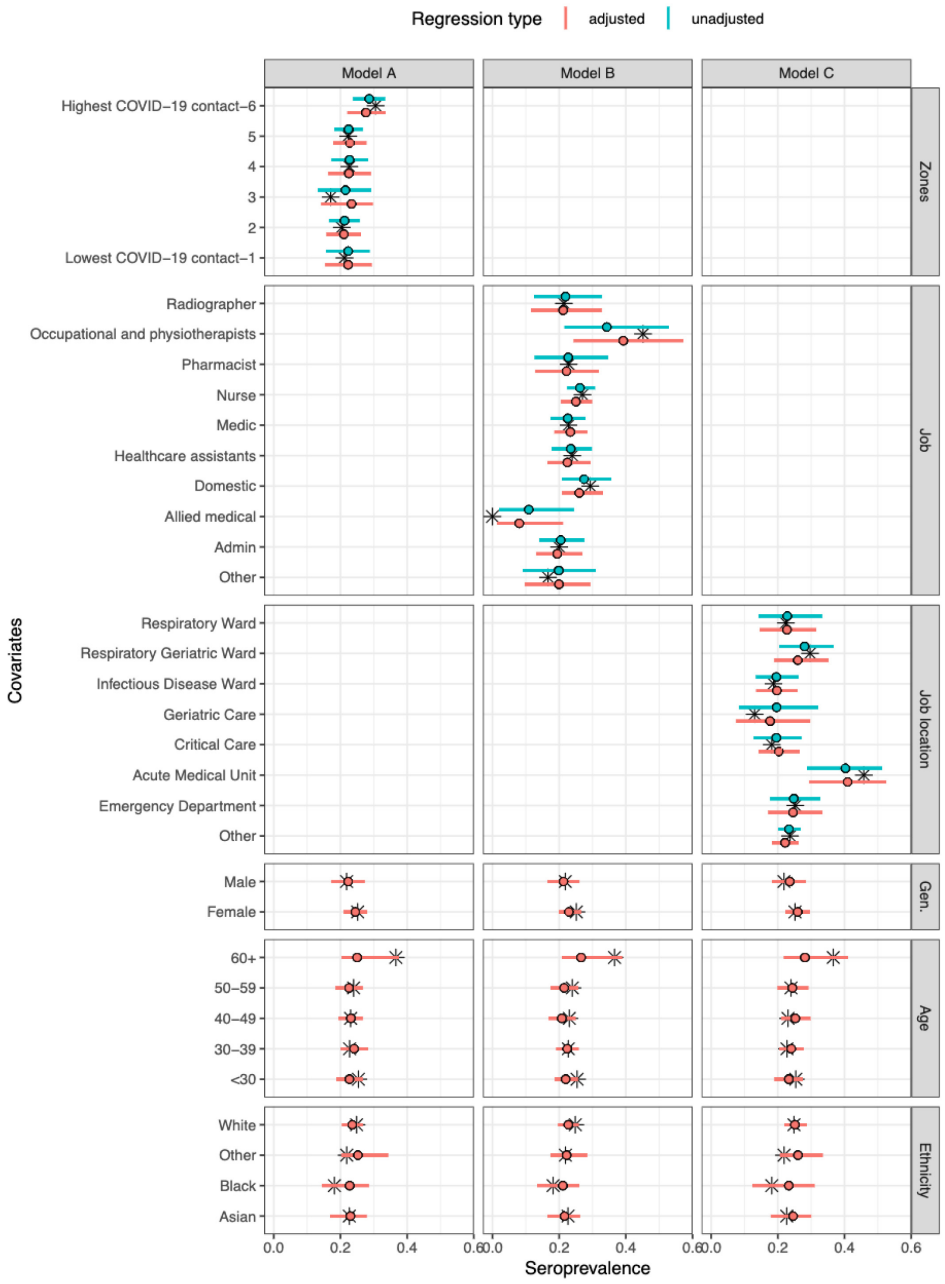
Model-predicted seroprevalence estimates for three different models (A-C), adjusted and unadjusted with covariates. Black stars represent point values from the data. The point and whiskers represent the mean value and 95% CrI of the posterior distribution. The three models differed by their primary exposure, Model A used COVID-19 zones (1 refers to lowest COVID-19 contact and 6 refers to highest COVID-19 contact ^21^.), Model B the job role, and Model C the job location. Each model was evaluated either unadjusted (primary exposure only) or adjusted (primary exposure with age, gender, and ethnicity).

The overall adjusted seroprevalence in the cohort was 23·1% (95% CrI 14·1–33·3), but varied across job type, job location and COVID-19 zone ( Figure 1; *Extended data:* Tables S3 to S7). A relatively high seroprevalence was seen in occupational and physiotherapists (39·2%, 95% CrI 24·4–56·5), and low seroprevalence in allied medical staff (9·2%, 95% CrI 1·4–21·3). Between wards, there was higher seroprevalence in the AMU (41·1%, 95% CrI 30·0–52·9) compared to other wards. Across COVID-19 zones, working in the areas with the highest degree of COVID-19 patient contact (zone 6) was associated with a slightly higher seroprevalence of 28·6% (95% CrI 24·0–33·5) compared to the other five groups ( Figure 1). The adjusted proportion of asymptomatic cases was 38·9% (95% CrI 23·6–57·3) ( *Extended data:* Figure S5). The proportion of asymptomatic cases remained relatively consistent across all covariates except for job type, where it ranged from 21·4% for occupational and physiotherapists up to 61·5% for domestic services staff ( *Extended data:* Figure S5).

### Antibody kinetics model

Differences in antibody concentration between samples were calculated for four different antibody-antigen interactions (spike-IgG, NCP-IgG, spike-IgA, NCP-IgA). Though there was a positive correlation between Spike-IgG and NCP-IgG across all samples (R ^2^.= 0·53), the correlations between serum IgG and IgA were much weaker (R ^2^. between 0·17 and 0·3) ( *Extended data:* Figure S6).

**Figure 2.**
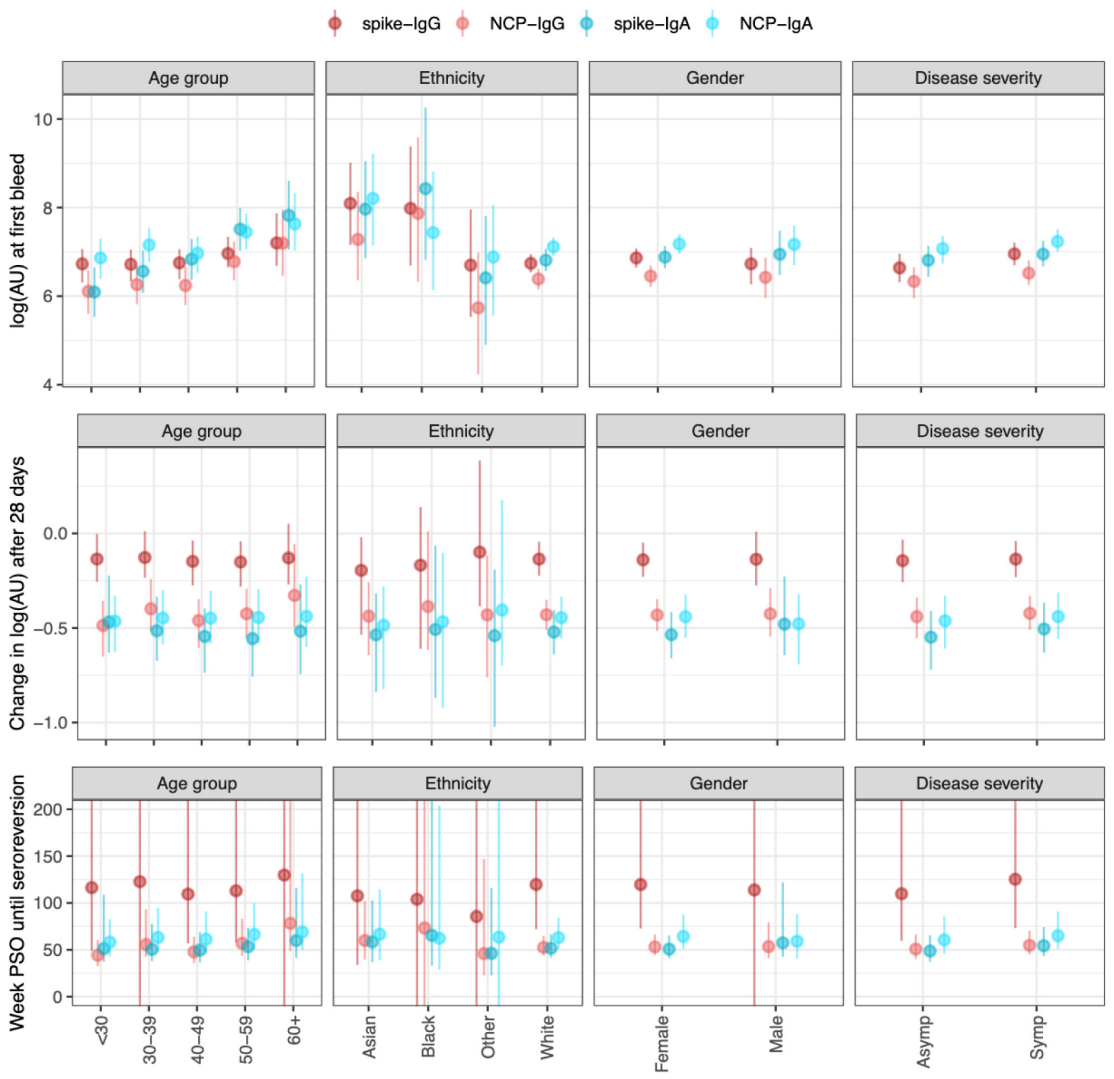
Outputs from the antibody kinetics model for four antibody-antigen interactions (spike-IgG, NCP-IgG, spike-IgA, and NCP-IgA). The IgG measures are in the WHO standard universal log2 antibody units, whereas the IgA measures are in log2(AU) units scaled relative to the values in the study. The dots show the median and the line segments show the 95% credible interval of the posterior distributions. Top panel shows the log2(AU) at the first bleed across four different covariates (Age group, ethnicity, gender, and disease severity). Middle panels show the change in log2(AU) after 30 days. The bottom panels show the time until seroreversion in weeks. Asymp (asymptomatic participants), Symp (symptomatic participants) PSO (post symptom onset).

For both serum IgG and IgA, older age groups showed higher antibody titres ( Figure 2); E.g., the median log2 titre of spike-IgG in those ≤30 years was 6·6 (95% CrI 6·2–7·0), and at 60+ years was 7·1 (95% CrI 6·6–7·8), while spike-IgA titre in those ≤30 years was 6·8 (95% CrI 6·3–7·3), and at 60+ years was 7·6 (95% CrI 7·0–8·3). Symptomatic cases showed similar titres compared to asymptomatic cases across IgG and IgA-serum measures. The reduction in antibody titre at the second blood draw was less in the spike-IgG (mean -0·15 (95% CrI -0·25– -0·06)) compared to NCP-IgG (mean -0·49 (95% CrI -0·58– -0·540)) and Spike-IgA/NCP-IgA. These estimated rates of decline remained consistent across all covariate groups studied. Consequently, the estimated time until seroreversion for seropositive samples from symptomatic participants was around 100 weeks for the spike-IgG, and 52 weeks for NCP-IgG and IgA serum measures ( Figure 2). When considering seropositive samples from symptomatic participants, there was little difference in the decrease in antibody levels as time post-symptom onset increased ( *Extended data:* Figure S7).

### Heterogeneous sensitivity model

**Figure 3.**
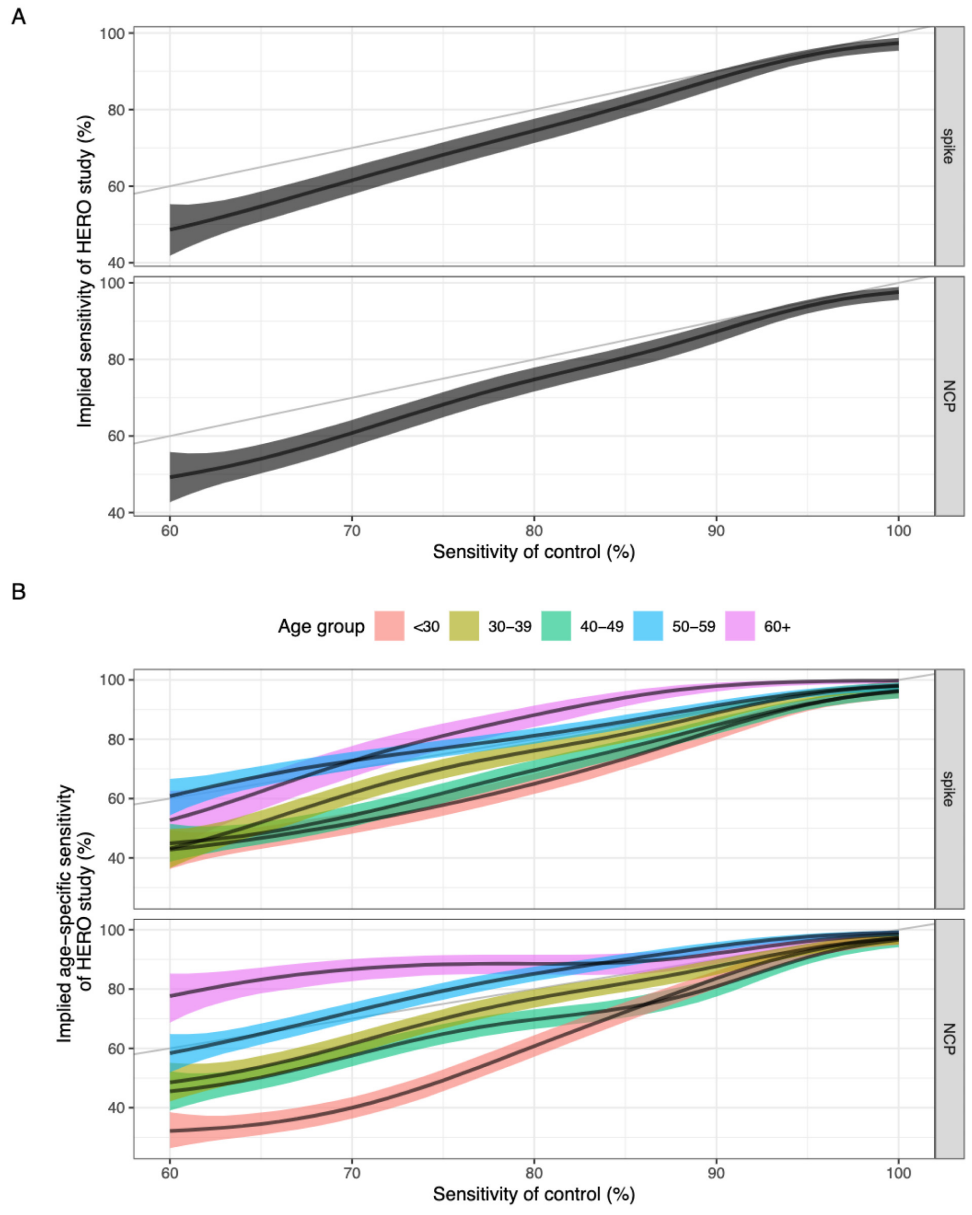
( **a**) Sensitivity of the assay validation dataset against the implied sensitivity of the HERO dataset for spike and nucleoprotein. ( **b**) Sensitivity of the assay validation dataset against the implied age-specific sensitivity in the HERO dataset for spike and nucleoprotein. Black line and ribbon shows median and 95% CrI for the posterior distributions respectively.

The heterogeneous sensitivity model demonstrates that using varying *A*_450_ cut-offs (corresponding to varying sensitivity values) to categorise seropositivity in the HERO dataset will result in a lower sensitivity than that defined using our assay validation dataset ( Figure 3a). The model also shows that there is no difference in implied sensitivity between using spike or NCP as the antigenic target in the ELISA assay.

The relationship between the *A*_450_ cut-off value and the sensitivity and specificity for the assay validation datasets for each antigen were plotted with the associated ROC curves ( *Extended data:* Figures S8 and S9). We hypothesised that the higher *A*_450_ values seen in older adults suggest that some commercially available serological assays may have a higher sensitivity in detecting COVID-19 antibodies in older age groups compared with younger age groups. We therefore used our model to estimate age-specific implied sensitivity for assays of different sensitivity profiles in estimating seroprevalence in our HERO dataset. We found that the sensitivity of a serological assay decreases with age due to the higher antibody titres seen in older people, with a clearer trend in an NCP-based assay compared to a spike-based assay ( Figure 3b). Assuming a theoretical assay validation set sample sensitivity of 80% for the NCP protein, the resulting median implied sensitivity for age groups <30, 30–39, 40–49, 50–59, and 60+ years was 61%, 77%, 70%, 85%, and 89% respectively.

## Discussion

We found a high SARS-CoV-2 seroprevalence in HCWs at a large UK hospital trust compared to national seroprevalence estimates, following the first pandemic wave in the UK ^28^. In addition, we identified important risk factors associated with occupational exposure to COVID-19, and described a significant association between age and the likelihood of a positive serological result which has important implications for the validation of SARS-CoV-2 antibody assays and the hitherto interpretation of population-level COVID-19 serology data.

Over 20% of HCWs at STH had evidence of SARS-CoV-2 infection within just over 100 days of the first confirmed COVID-19 patient being admitted to our NHS trust. This high proportion over a short space of time is likely representative of the much higher exposure to SARS-CoV-2 infection among certain subpopulations of the workforce that we tested. Although data from other settings and countries suggested infection risk in HCWs is similar to community exposure, this seroprevalence is much higher than estimated seropositivity in the UK population at a similar time (6·0%, 95 CrI 5·8–6·1 in July 2020) ^28, 29^.

Our data show that HCWs working in AMUs are at significantly higher risk of infection with SARS-CoV-2, with seropositivity rates above that of other wards, consistent with other UK studies ^5, 6, 30^. EDs face a similar patient turnover yet have lower HCW seroprevalence rates in both ours and previous reports ^5, 6, 30, 31^. Although some patient factors may increase HCW risk of infection on AMU compared to ED (cohort bays, longer stays, more fomites e.g. bedside tables, chairs), more frequent interactions and therefore transmission between HCWs may also play a significant additional role. In the event of a further wave or outbreak, infection prevention and control (IPC) interventions to reduce risk in these areas could include targeted IPC training and auditing (particularly of PPE use and break areas), serial staff testing, pop-up isolation units in bay areas and optimising staff-to-patient ratios. At the other end of the spectrum, we and others have found that HCWs in critical care units have some of the lowest seropositivity rates, which likely reflects ‘Level 3 Airborne’ PPE use in Critical Care units from an early point in the pandemic and the increased availability of negative pressure rooms ^5, 6, 8, 30, 31^.

Occupational and physiotherapists (OT/PT) had the highest rates of seroprevalence across all of the job roles included in our cohort (45.5%), which is consistent with some other UK studies ^6, 32^. OT/PT work involves prolonged close contact with patients in addition to PT performing chest physiotherapy and open suctioning of the respiratory tract. In addition, OT/PT work across multiple inpatient and outpatient areas in our Trust, which could increase risk of transmission from both patients and other HCWs.

Increasing age was associated with seropositivity, with over a third of our HCWs aged >60 testing seropositive, and with higher antibody titres. We demonstrate that the sensitivity of a serological assay increases with increasing age due to the higher antibody titres seen in older people, and with a clearer trend in NCP- compared to spike-based assays. Consistent with other studies, we also found that NCP-IgG is likely to wane more quickly than Spike-IgG. Depending on the sampling time frame relative to pandemic wave, therefore, serological testing based on NCP-IgG alone may therefore underestimate seroprevalence i.e. potential exposure to infection. As two of the major commercial SARS-CoV-2 antibody assays (Roche Elecsys and Abbott SARS-CoV-2 IgG) were validated with patient sera collected from those with more severe disease early on in the pandemic (i.e. those who presented to health services). Previous studies clearly demonstrate that patients with more severe COVID-19 have higher antibody titres and it would be reasonable to assume these cases were likely to also be older in age ^33– 36^. Our findings suggest that using such samples collected from severely symptomatic older patients for the purposes of assay calibration may result in an assay with lower or insufficient sensitivity when applied to less symptomatic or younger (often community) populations. With increasing vaccine coverage, use of spike IgG to determine seroprevalence also becomes more problematic when distinguishing whether an individual is seropositive from vaccination or previous infection. Assays which combine antibody responses to membrane protein with NCP antibodies may overcome these challenges ^37, 38^.

We note the limitations of our study, which include a potential for selection bias due to participants self-enrolling for convenience, rather than using systematic sampling. Reassuringly, our seroprevalence rates are similar to those seen in other UK based seroprevalence studies ^5, 32^. In addition, we recognise that our cohort has relatively low numbers of HCWs from minority ethnic backgrounds (~10%), compared to the Sheffield general population (19%) ^39^.

With the ongoing global devastation caused by the COVID-19 pandemic and its lasting effect on healthcare services, understanding the risk factors leading to HCW exposure is paramount to ensuring the continuity of effective and safe patient care. Our real-world data suggest that NHS HCWs face high levels of exposure to SARS-CoV-2, plus highlights locations and job roles at greatest risk during the first wave of the pandemic. Population seroprevalence data can help guide decision makers on risk management. Using assays that have been validated using serum samples from a broad population, combined with antibody kinetic modelling and/or with age-adjusted cut-offs could overcome the potential limitations we have highlighted.

## Data availability

### Underlying data

Zenodo: dchodge/hero-study: Submitted version for WOR, https://doi.org/10.5281/zenodo.5215671 ^26^.

The project contains the following underlying data:

- datafit.RData
- fit_mcmc_asymp.RData
- fit_mcmc_change.RData
- fit_mcmc_prev.RData
- fit_mcmc_sens.RData
- fit_mcmc_spec.RData
- fit_mcmc_start.RData
- fit_mcmc_time.RData
- sensspec.RData
- sero_clean.RData

### Extended data

Zenodo: dchodge/hero-study: Submitted version for WOR, https://doi.org/10.5281/zenodo.5215671 ^26^.

The project contains the following extended data:

   –   Table S1. Details of the samples used to set thresholds during assay validation
   –   Table S2. Comparison of antibody units in assay calibration curve sera assigned to the assay with WHO international standard antibody units
   –   Table S3. Summary of the response variables and the covariates used in the regression model.
   –   Table S4. Summary of the model parameters used in the regression model.
   –   Table S5. Summary of the response variables and the covariates used in the regression model.
   –   Table S6. Summary of the model parameters used in the regression model.
   –   Table S7. Summary of the model parameters used in the Heterogenous sensitivity model.
   –   Figure S1. ROC curves of the spike and NCP assays
   –   Figure S2. Comparison of IgA assay A450 based on IgG Serostatus
   –   Figure S3. Study flow diagram.
   –   Figure S4. Histogram (overlayed) showing the symptom onset, date of first bleed (all cases and symptomatic cases only), and time at second bleed (all cases and symptomatic cases only).
   –   Figure S5. Model-predicted proportion of asymptomatic estimates for three different models (A-C), adjusted and unadjusted with covariates gender, age group and ethnicity.
   –   Figure S6. Correlation between the four different antibody measures for 264 serological samples.
   –   Figure S7. Rate of decline for the antibody concentrations post-symptom onset for the four antibody measures. The fitted line is from a linear regression, with the 95% CI shown in red.
   –   Figure S8. Relationship between sensitivity/specificity and the cutoff value for the control dataset.
   –   Figure S9. ROC curves with the *A*_450_ cut-off value indicated in red for the control dataset. x-axis shows the False Positive Rate, y-axis is the sensitivity.
   –   Figure S10. ROC curves for different age groups and antigen proteins, with the *A*_450_ cut-off value indicated in various colours for the control dataset.
   –   Figure S11. (a) Specificity of the control data set against the implied specificity of the HERO dataset for spike and nucleoprotein. (b) Specificity of the control data set against the implied age-specific specificity of the HERO dataset for spike and nucleoprotein.
   –   HERO Completed STROBE Checklist[60].doc

Data are available under the terms of the Creative Commons Attribution 4.0 International license (CC-BY 4.0).
