# Supplementary material for: Risk factors for SARS-CoV-2 seroprevalence following the first pandemic wave in UK healthcare workers in a large NHS Foundation Trust
Source: Wellcome Open Res. 2022 Jun 10;6:220. Originally published 2021 Sep 3. [Version 3] doi: 10.12688/wellcomeopenres.17143.3 (PMC9091808; doi:10.12688/wellcomeopenres.17143.3)
Supplement: Peer review history

# Peer review: Reviewer response for version 2

**DOI:** 10.21956/wellcomeopenres.19649.r50270

**Published:** 2022-05-10

**Authors:** Patrick Lillie

**Affiliations:** 1 Hull University Teaching Hospitals NHS Trust, Hull, UK; 2 Hull York Medical School, Heslington, UK

This is an interesting paper and the authors have done a very thorough job. Obviously, with the progression of the pandemic, vaccination roll out and development of subsequent variants these data may not have the same relevance as during the first wave. I have a few specific points, but nothing too critical to the article.

 As both spike and nucleocapsid antibodies were tested for vaccination is less of an issue, but as the recruitment period in the study overlapped with vaccine trials that were recruiting, it would be worth clarifying that none of the participants were in those early vaccine trials.

 What case definition was used for the symptomatic definition? Was there any major difference in the seroprevalence between those who had classical COVID-19 symptoms and those with more viral respiratory type symptoms?

 It would be really helpful if there were some data on local community prevalence/seropositivity around the testing dates (I appreciate that the UK figure of 4% is given for April 2020 but local data would be helpful in assessing the numbers).

 Was there a difference in PPE use that could explain the difference in seroprevalence between ED and AMU? Many ED's retained use of higher level respiratory PPE due to performing aerosol generating procedures, which would align it with critical care areas.

Is the work clearly and accurately presented and does it cite the current literature?

Yes

If applicable, is the statistical analysis and its interpretation appropriate?

I cannot comment. A qualified statistician is required.

Are all the source data underlying the results available to ensure full reproducibility?

Yes

Is the study design appropriate and is the work technically sound?

Yes

Are the conclusions drawn adequately supported by the results?

Yes

Are sufficient details of methods and analysis provided to allow replication by others?

Yes

Reviewer Expertise:

Clinical infectious diseases, vaccine trials,

I confirm that I have read this submission and believe that I have an appropriate level of expertise to confirm that it is of an acceptable scientific standard.

# Response

**Published:** 2022-06-07

**Authors:** Hayley Colton

**Affiliations:** Sheffield Teaching Hospitals Nhs Foundation Trust, UK

Thank you for taking the time to review our paper. We’ve responded to your points below: 

1. We were mindful of participation in vaccine trials affecting our results, as we were involved with vaccine trials locally. Following their V1 visit, five of our participants had subsequently enrolled (and seroconverted) after taking part in a vaccine trial, so were excluded from V2 analysis. This is not mentioned explicitly in the manuscript but is stated in Supplementary Figure 3 "Study Flow Diagram" in Extended Data (reference 24 in the manuscript). This has been added to the manuscript: " *1174 attended a second visit (V2) between 15 June and 10 July 2020, **however eight samples were excluded from the V2 analysis as they were either unlabelled (n=2), lost (n=1) or the participant had participated in a COVID-19 vaccine trial (and ***seroconverted)***  (n=5)** ( Extended data:* *Figures S3 and S4).*"
2. We did not directly compare classical COVID symptoms vs other viral respiratory symptoms within this study. Participants self reported any previous possible or confirmed COVID-19 episodes on the electronic questionnaire (the wording of all of the categories is on page 3 of Extended data, reference 24 in the manuscript and also below), and then we compared asymptomatic vs symptomatic groups within the results. Beyond the symptomatic / asymptomatic definition, all the other symptom 'questions' asked were about those that had been specifically cited as being associated with COVID-19 at the time, before such a thing as 'classical' COVID-19 was defined - so any comparison between 'classic COVID and 'other' viral symptoms would be post hoc.
  "The certainty of an illness being consistent with COVID-19 was classified as:  
  - Diagnosed COVID-19 and NAAT confirmed  (“YES - I was diagnosed and it was confirmed with a swab test”),
  - Diagnosed COVID-19 but NAAT not performed (“YES - I was diagnosed but it has not been confirmed with swab test”),  
  - Self reported Symptomatic  (“MAYBE - I've had symptoms of cough, fever or headache or required days of work for a new medical problem”),  
  - Asymptomatic  (“NO” or “NO and I’ve had a negative PCR test”)" 
3. I was unable to find any specific seroprevalence data for Yorkshire & The Humber for around April 2020, however seroprevalence of blood donors in the North East (which includes Yorkshire & The Humber), in Mid April was approximately 3.5%, which is similar to the estimation used in the analysis (Weekly Coronavirus Disease 2019 (COVID-19) Surveillance Report Week 22 Year 2020).  
4. Good point - I have reviewed our internal records and found that ED implemented a suspected COVID-19 cohort area on 19 ^th^ March 2020, where level 2 airborne PPE was advised in cohort areas, which may have contributed towards lower levels of seropositivity when compared to AMU. This has been added to the discussion: *"* *EDs face a similar patient turnover yet have lower HCW seroprevalence rates in both ours and previous reports ^5, 6, 35, 36 ^. **One factor contributing to the difference seen between ED and AMU, may be that our ED implemented a suspected / confirmed COVID-19 cohort area on 19 ^th^ March 2020, where level 2 airborne PPE was mandated.** **In addition, on AMU there were likely** longer stays of asymptomatic/paucisymptomatic cases prior to universal PPE use, more fomites (e.g. bedside tables, chairs), ****and more frequent interspeciality interactions resulting in transmission between HCWs* ^43^
  Ref 43- Lindsey et al. Characterising within-hospitalSARS-CoV-2 transmission events using epidemiological and viral genomic data across two pandemic waves. Nat Commun. 2022 Feb 3;13(1):671."

# Peer review: Reviewer response for version 2

**DOI:** 10.21956/wellcomeopenres.19649.r49137

**Published:** 2022-03-16

**Authors:** Mo Yin

**Affiliations:** 1 Mahidol Oxford Tropical Medicine Research Unit, Faculty of Tropical Medicine, Mahidol University, Bangkok, Thailand; 2 National University of Singapore, Singapore, Singapore

Thank you for the extensive effort in addressing my earlier queries. I have no further comments.

Is the work clearly and accurately presented and does it cite the current literature?

Partly

If applicable, is the statistical analysis and its interpretation appropriate?

Partly

Are all the source data underlying the results available to ensure full reproducibility?

Yes

Is the study design appropriate and is the work technically sound?

Partly

Are the conclusions drawn adequately supported by the results?

Partly

Are sufficient details of methods and analysis provided to allow replication by others?

Partly

Reviewer Expertise:

Mathematical modelling, data analysis, trial designs, randomised controlled trials

I confirm that I have read this submission and believe that I have an appropriate level of expertise to confirm that it is of an acceptable scientific standard.

# Peer review: Reviewer response for version 1

**DOI:** 10.21956/wellcomeopenres.18936.r47144

**Published:** 2021-12-06

**Authors:** Mo Yin

**Affiliations:** 1 Mahidol Oxford Tropical Medicine Research Unit, Faculty of Tropical Medicine, Mahidol University, Bangkok, Thailand; 2 National University of Singapore, Singapore, Singapore

I congratulate the authors for conducting this large and informative study. It is an important finding that SARS-CoV-2 were highly prevalent amongst healthcare workers so rapidly into the pandemic.

 My overall comments are:

1. Figure 1 shows that the association between high seroprevalence and age attenuated after adjustment with zones, job and wards. Could there be confounding relationships? If zones, job and wards were added to the antibody kinetics model, would the association between age and high titre attenuate as well?
2. It appears that a major aim here is to valid an in-house assay. The manuscript could be more clear if this was clearly stated in the beginning. An important caveat for this work is that the dataset was based on this in-house assay.

My complete list of comments are listed below: 

**Abstract:**

1. “SARSCoV- 2 antibodies were tested using an in-house assay for IgG and IgA reactivity against Spike and Nucleoprotein (sensitivity 99·47%, specificity 99·56%).” This was in the results section of the manuscript. Was validation of the in-house assay an objective of this work? If yes this should be clearly specified and the quoted text should go into results.
2. “HCWs in acute medical units working closely with COVID-19 patients were at highest risk of infection, though whether these are infections acquired from patients or other staff is unknown.” I would assume HCWs from other ward types also work closely with patients?
3. “Current serological assays may underestimate seroprevalence in younger age groups if validated using sera from older and/or more symptomatic individuals.” It was not shown in the results that symptomatic individuals had higher titres than asymptomatic ones?

**Introduction:**

1. “The true number of HCWs exposed to SARS-CoV-2 to-date is unknown, particularly during the early stages of the pandemic. Initial methods to estimate HCW exposure included extrapolation from work absenteeism rates, and are unlikely to be reliable3. Confirmation by molecular testing increased the accuracy of case detection, although access to nucleic acid amplification testing (NAAT) was limited during the early stages of the pandemic in the UK4. Detection of exposure by antibody seroconversion may provide a more accurate estimate of risk in HCW populations, can be performed at large scale, and is less affected by symptom activated testing pathways5–8.”  Do the authors mean infected/infection instead of exposed/exposure since confirmation testing by NAAT refers to infection rather than exposure?
2. “Many antibody assays have been evaluated using samples from hospitalised patients; it is unclear how these assays perform with the lower antibody levels found in those with more mild or asymptomatic SARS-CoV-2 infection10,12” There have been a number of studies looking at serology response in mild COVID cases, e.g. https://www.nature.com/articles/s41598-020-77125-8. ^1^ Suggest to include in the discussion how the findings from this study compare to the existing ones.
3. Please specify the aims in the introduction.

**Methods:**

1. Please include more details on the ward set ups in the ‘Background and setting’ for readers not familiar with the NHS. There are some descriptions in the results and discussion sections. It is probably clearer if these can be explained upfront.
2. It appears that willing healthcare workers enrolled themselves into the study. Were there any measures to prevent volunteer bias? Could volunteer bias influence the results?
3. “These were categorised as: i), diagnosed with COVID-19 and confirmed by NAAT, ii), clinically diagnosed with COVID-19 but NAAT not performed, and iii), self-reported symptoms only21. Together, we defined these three groups as “symptomatic”, as asymptomatic testing was only introduced after the study recruitment period.” If we expect a proportion of those in groups ii) and iii) are not true COVID-19 cases, how would that affect the results? Were any sensitivity analyses performed to address this uncertainty?
4. “Using seropositivity as the binary response variable, we considered three different model subtypes with varying primary exposures; job location, contact with COVID-19 patients, and job type21.” It is not clear from this sentence what were the actual independent variables used in the model subtypes? Which variable was given a random effect in the multilevel model? In the GitHub account, where the README says ‘The stan code and the raw mcmc outputs is contained in the outputs/ folder.’, I could not find the model codes?
5. “For the antibody kinetics model, we included samples from seropositive individuals in a Bayesian multilevel linear regression model in two parts:…” Does this refer to the HCWs who were positive in the first blood draw only?
6. “In our heterogeneous sensitivity and specificity model, we explored how estimates derived from our assay validation dataset generalise to covariate groups, e.g. participant age.” What is the assay validation dataset? It is probably clearer to move the explanation from results to the methods section.
7. “In our heterogeneous sensitivity and specificity model, we explored how estimates derived from our assay validation dataset generalise to covariate groups, e.g. participant age. To model the generalisability of these performance measures, we compared the seropositivity classification of our study dataset using our in-house antibody assay, with the predicted seropositivity classification from hypothetical assays with a quoted sensitivity and specificity. Our” These sentences are a little confusing. Firstly, what does ‘estimates’ in ‘estimates derived from our assay validation dataset generalise to covariate groups, e.g. participant age’ refer to? Is it referring to sensitivity and specificity? Secondly, “we compared the seropositivity classification of our study dataset using our in-house antibody assay” sounds like you compared the classification using the antibody assay. Thirdly, in “with the predicted seropositivity classification from hypothetical assays with a quoted sensitivity and specificity”, what does ‘quoted’ mean? Does it mean ‘assumed sensitivity and specificity since it is a hypothetical assay?
8. Please introduce A450 when it was first mentioned in the manuscript.
9. In “to model how reliably quoted performance measures generalise.”, what does ‘quoted’ refer to?
10. “Using the assay validation dataset, we estimated the A450 cut-off value for a range of chosen sensitivity values, and then used this A450 cut-off to classify seropositivity in the study dataset.” How did you choose a single A450 value from a range of sensitivity values?
11. “We then estimated the implied sensitivity on the HERO dataset by comparing seropositivity classification based on the estimated A450 cut-off value, with the seropositivity classification from our in-house assay (which for ease of comparison, we assume represents the maximum possible sensitivity and specificity (i.e. 100%) in this model).” What does ‘implied’ mean here?
12. Please cite R and cmdstanr.

**Results:**

1. “Rapid waning of IgA responses following SARS-CoV-2 infection complicated defining positive and negative samples based on the convalescent sera we used for assay validation.” Why is this so?

1. “1174 attended for a second visit (V2) between 15 June and 10 July 2020” ‘for’ is unnecessary in this sentence.
2. Figure 1: Why do the black stars not overlap with the unadjusted mean estimates?
3. Why were zones, job and wards not used in the same model? Was their model selection done?
4. Figure 1: The raw data showed that a higher proportion of HCWs who were >60 years old were seropositive compared to the other age groups. However, there’s no longer this association after adjustment. What was/were the confounder(s)?
5. In the antibody kinetics model, neither zones, job and wards were used as an independent variable. As mentioned in the earlier point, would this finding of age and higher titres modulate/disappear if zones, job and/or wards are included in this model as well?
6. Figure 3: Please specify the meaning of ‘control’ in the x-axis.

**Discussion:**

1. Were the infection prevention and control measures similar in the ED and AMU? Could the higher prevalence in AMU be due to transmissions from asymptomatic/ mildly symptomatic patients who were not triaged to be isolated by the ED?
2. “Previous studies clearly demonstrate that patients with more severe COVID-19 have higher antibody titres” However this was not found in this study?
3. “Reassuringly, our seroprevalence rates are similar to those seen in other UK based seroprevalence studies” Citation 5 also enrolled volunteer participants (publicised study using social media), and citation 21 is the HERO extended data?
4. It will be informative to have a more detailed discussion on if the finding of age affecting serology titres were reported in other studies.

Is the work clearly and accurately presented and does it cite the current literature?

Partly

If applicable, is the statistical analysis and its interpretation appropriate?

Partly

Are all the source data underlying the results available to ensure full reproducibility?

Yes

Is the study design appropriate and is the work technically sound?

Partly

Are the conclusions drawn adequately supported by the results?

Partly

Are sufficient details of methods and analysis provided to allow replication by others?

Partly

Reviewer Expertise:

Mathematical modelling, data analysis, trial designs, randomised controlled trials

I confirm that I have read this submission and believe that I have an appropriate level of expertise to confirm that it is of an acceptable scientific standard, however I have significant reservations, as outlined above.

## References

[1] : Serological follow-up of SARS-CoV-2 asymptomatic subjects. *Scientific Reports* .2020;10(1) : 10.1038/s41598-020-77125-8 10.1038/s41598-020-77125-8 33208819PMC7674414

# Response

**Published:** 2022-03-01

**Authors:** Hayley Colton

**Affiliations:** Sheffield Teaching Hospitals Nhs Foundation Trust, UK

Thank you to the reviewer for their considerate and thorough comments. Please see below our responses to each point, and changes made.

 1. Figure 1 shows that the association between high seroprevalence and age attenuated after adjustment with zones, job and wards. Could there be confounding relationships? If zones, job and wards were added to the antibody kinetics model, would the association between age and high titre attenuate as well?

**David Hodgson: The reviewer’s interpretation of Figure 1 seems correct, but to be crystal clear; Figure 1 shows the (unadjusted) association between high seroprevalence, and the covariates age, gender, and race, and the (adjusted) association between high seroprevalence, and the covariates age, gender, race, and one primary exposure variable (either zones, jobs or wards). In Figure 1, the similarity between the unadjusted and adjusted seroprevalence estimates suggest that neither zone, jobs or wards were strong confounders on the age-dependent prevalence, therefore they are dropped in the subsequent modes for parsimony.**

*2. *It appears that a major aim here is to valid an in-house assay. The manuscript could be more clear if this was clearly stated in the beginning. An important caveat for this work is that the dataset was based on this in-house assay.

**Hailey Hornsby: As seen later in this response, the manuscript intro has been expanded to include the ELISA validation as an objective of the work:**

**“ To achieve this, we sought to measure SARS-CoV-2 antibody titres by developing an in-house assay (...)”**

 Abstract:

*1. “SARSCoV- 2 antibodies were tested using an in-house assay for IgG and IgA reactivity against Spike and Nucleoprotein (sensitivity 99·47%, specificity 99·56%).” *This was in the results section of the manuscript. Was validation of the in-house assay an objective of this work? If yes this should be clearly specified and the quoted text should go into results.

**Hailey Hornsby / Hayley Colton: Thank you for pointing out, this has now been corrected:**

**“Methods: HCWs at Sheffield Teaching Hospitals NHS Foundation Trust were prospectively enrolled and sampled at two time points. We developed an in-house ELISA for testing participant serum for SARS-CoV-2 for IgG and IgA reactivity against Spike and Nucleoprotein. Data were analysed using three statistical models: a seroprevalence model, an antibody kinetics model, and a heterogeneous sensitivity model. Results: Our in-house assay had sensitivity 99·47% and specificity 99·56%. We found that 24·4% (n=311/1275) of HCWs were seropositive as of 12th June 2020.”**

 2. *“HCWs in acute medical units working closely with COVID-19 patients were at highest risk of infection, though whether these are infections acquired from patients or other staff is unknown.” *I would assume HCWs from other ward types also work closely with patients?

**Hayley Colton: HCWs were asked in the survey what zone they worked in, and if they were patient facing (see Extended Data, reference 21). The analysis showed that the highest seroprevalence was seen in AMU and those who worked in red zones regularly in close contact with COVID patients (group 6). Thank you for pointing out that the wording is not clear, it has been changed in the manuscript:**

**“HCWs in acute medical units and those working closely with COVID-19 patients were at highest risk of infection, though whether these are infections acquired from patients or other staff is unknown”**

 3. *“Current serological assays may underestimate seroprevalence in younger age groups if validated using sera from older and/or more symptomatic individuals.” *It was not shown in the results that symptomatic individuals had higher titres than asymptomatic ones?

**Hayley Colton: Commercial assays referred to in this sentence were validated against samples from patients who were hospitalised with severe disease; whereas our assay was validated using samples from both inpatients with severe disease and outpatients with mild (or no) symptoms). Although we found that symptomatic cases showed similar titres compared to asymptomatic cases within our HCWs, we did not analyse the titres against severity, whereas other studies have. For our assay development, we did compare inpatient and outpatient antibody titres, this has now been added to the Extended Data as Figure S1b. Thank you for pointing this out, the manuscript has been amended to reflect that we are referring to severity rather than symptoms exclusively:**

**“Current serological assays may underestimate seroprevalence in younger age groups if validated using sera from older and/or more severe COVID-19 cases.”**

 Introduction:

 1. " *The true number of HCWs exposed to SARS-CoV-2 to-date is unknown, particularly during the early stages of the pandemic. Initial methods to estimate HCW exposure included extrapolation from work absenteeism rates, and are unlikely to be reliable3. Confirmation by molecular testing increased the accuracy of case detection, although access to nucleic acid amplification testing (NAAT) was limited during the early stages of the pandemic in the UK4. Detection of exposure by antibody seroconversion may provide a more accurate estimate of risk in HCW populations, can be performed at large scale, and is less affected by symptom activated testing pathways5–8.”  *Do the authors mean infected/infection instead of exposed/exposure since confirmation testing by NAAT refers to infection rather than exposure?

**Hayley Colton: Thank you for pointing out, rephrased as below:**

**“The true number of HCWs infected with SARS-CoV-2 to-date is unknown, particularly during the early stages of the pandemic. Initial methods to estimate HCW COVID-19 cases included extrapolation from work absenteeism rates, and are unlikely to be reliable3. Confirmation by molecular testing increased the accuracy of case detection, although access to nucleic acid amplification testing (NAAT) was limited during the early stages of the pandemic in the UK4. Serological testing can be performed at large scale, and is less affected by symptom activated testing pathways, so may provide a more accurate estimate of previously infected HCWs and could be used in conjunction with other data to determine their risk factors for exposure 5–8.”**

 2. *"Many antibody assays have been evaluated using samples from hospitalised patients; it is unclear how these assays perform with the lower antibody levels found in those with more mild or asymptomatic SARS-CoV-2 infection10,12”* There have been a number of studies looking at serology response in mild COVID cases, e.g. https://www.nature.com/articles/s41598-020-77125-8. 1 Suggest to include in the discussion how the findings from this study compare to the existing ones.

**Hayley Colton: Thank you, this didn’t come up in our original literature search. It appears Wantai ELISA which is used in the UNICORN consortium reference was also validated using sera from samples from PCR positive patients who all had clinical symptoms, laboratory abnormalities or pulmonary imaging manifestations. (WANTAI SARS-CoV-2 Ab ELISA Instructions for use https://www.fda.gov/media/140929/download accessed 30 Dec 2021). I have added this to the introduction and discussion.**

**“ Several commercial SARS-CoV-2 antibody assays have been validated using samples from patients with more severe COVID-19, and some studies have suggested those with milder or asymptomatic COVID-19 are less likely to develop detectable antibodies. 40, 41, 42.”**

 3. Please specify the aims in the introduction.

**Hayley Colton: We have edited the manuscript introduction to make our aims clearer:**

**“In this study we aimed to investigateSARS-CoV-2 seroprevalence in HCWs at Sheffield Teaching Hospitals NHS Foundation Trust (STH) following the first way of the pandemic in the UK. T o achieve this, we sought to measure SARS-CoV-2 antibody titres by developing an in-house assay, and use statistical modelling to explore risk factors associated with seropositivity, the evolving antibody response, and the impact of age on assay sensitivity”**

 Methods:

 1. Please include more details on the ward set ups in the ‘Background and setting’ for readers not familiar with the NHS. There are some descriptions in the results and discussion sections. It is probably clearer if these can be explained upfront.

**Hayley Colton:  Thanks for pointing this out, the background section has been edited:**

**“STH is an NHS trust offer ing secondary- and tertiary-level care across four sites in South Yorkshire, UK, with 1,669 inpatient beds and 18,500 employees 20. Patients with a medical reason for admission are typically admitted to STH either through attending the Emergency Department (ED) or through referral to the Acute Medical Unit (AMU) by their General Practitioner (GP). On AMU, patients are given an initial management plan then triaged to the most appropriate medical speciality ward e.g. respiratory medicine. The first patient with confirmed COVID-19 was admitted to STH on 23 February 2020; the first wave of the UK pandemic occurred between March 2020 and June 2020. Patients with suspected or confirmed COVID-19 were referred directly to the infectious diseases wards by GPs or from STH admission units. When infectious diseases capacity was reached, suspected COVID-19 patients would either be placed in side rooms or cohorted in bays, whilst confirmed COVID-19 patients could be moved to COVID cohort wards”**

 2. It appears that willing healthcare workers enrolled themselves into the study. Were there any measures to prevent volunteer bias? Could volunteer bias influence the results?

**Paul Collini: Volunteer bias could conceivably increase the observed prevalence if those who thought they had been exposed to COVID were more likely to volunteer and really had been more likely to have been exposed and infected. The only measure we had to prevent bias was that we approached domestic services staff through their managers as well as email as we anticipated they wouldn’t be accessing emails. However, we had no measures to mitigate any volunteer enrolment bias. While we cannot measure the extent of this effect on the measured prevalence of antibody detection, we suspect that it is small; at the time of the study there was a huge interest among staff as to whether they had yet been exposed to SARS CoV-2, which appeared to be irrespective of perceived exposure and was reflected in the rapid enrolment; also at that time it was still not appreciated how common asymptomatic COVID was, yet a large number of people without any prior symptoms enrolled. Even if volunteer bias were to artificially increase the observed prevalence, we think any volunteer bias would have been equal across all categories we compared, and so not altering the validity of these comparisons. We have altered the discussion to better reflect this - see response to ‘Discussion point 3’.’**

 3. *“These were categorised as: i), diagnosed with COVID-19 and confirmed by NAAT, ii), clinically diagnosed with COVID-19 but NAAT not performed, and iii), self-reported symptoms only21. Together, we defined these three groups as “symptomatic”, as asymptomatic testing was only introduced after the study recruitment period.” *If we expect a proportion of those in groups ii) and iii) are not true COVID-19 cases, how would that affect the results? Were any sensitivity analyses performed to address this uncertainty?

**David Hodgson: Just to be clear, case positivity was defined through seropositivity and this sentence refers to the classification of these seropositive individuals as either a symptomatic or asymptomatic COVID-19 case. For patients in group ii) the COVID case was diagnosed by a clinical professional but a NAAT test was not possible at the time. Therefore it is unlikely, especially given the short time-scale between reporting COVID-like symptoms (Feb 2020) and the first bleed of the experiment (Apr 2020) that this is a misdiagnosed case. For people in iii) it is possible that some people were asymptomatic with COVID-19 and were reporting symptoms of another respiratory disease, however the sample size of this group is small (22 / 340 (6.4%) of non-asymptomatic cases) and even if such misdiagnoses were common, it is unlikely to impact the results.**

 4. *"Using seropositivity as the binary response variable, we considered three different model subtypes with varying primary exposures; job location, contact with COVID-19 patients, and job type21.” *It is not clear from this sentence what were the actual independent variables used in the model subtypes? Which variable was given a random effect in the multilevel model? In the GitHub account, where the README says ‘The stan code and the raw mcmc outputs is contained in the outputs/ folder.’, I could not find the model codes?"

**David Hodgson: Further details of the model are given in the Extended Data (reference 21). To make the model independent variables as clear as possible in manuscript, we have edited the manuscript as below:**

**“Using seropositivity as the binary response variable, we considered three different Bayesian Hierarchical Regression model subtypes all with explanatory demographic variables age, race and gender, and each model with a different primary exposure; job location, contact with COVID-19 patients, and job type21. ”**

**Regarding the stan code and raw mcmc outputs; there is a typo in the README file, and these files are actually contained in the ‘include’ folder. This has now been updated in the Github Repository. Thankyou for pointing out this error.**

 5. *"For the antibody kinetics model, we included samples from seropositive individuals in a Bayesian multilevel linear regression model in two parts:…” *Does this refer to the HCWs who were positive in the first blood draw only?

**David Hodgson: We included HCWs who were positive at both their first and second bleed as stated in the Extended Data (reference 21 in the manuscript) Manuscript edited as following:**

**“For the antibody kinetics model, we included samples from individuals who were seropositive at both bleeds, in a Bayesian multilevel linear regression model in two parts:”**

 6. *“In our heterogeneous sensitivity and specificity model, we explored how estimates derived from our assay validation dataset generalise to covariate groups, e.g. participant age.” *What is the assay validation dataset? It is probably clearer to move the explanation from results to the methods section.

**Hayley Colton: The validation of the in-house assay has now been added as an objective:**

**“T o achieve this, we sought to measure SARS-CoV-2 antibody titres by developing and using an in-house assay, prior to using statistical modelling to explore risk factors associated with seropositivity, the evolving antibody response, and the impact of age on assay sensitivity”**

**The development technique of the in-house assay has been moved to methods, whilst the validation results have been moved to results.**

 7. *"In our heterogeneous sensitivity and specificity model, we explored how estimates derived from our assay validation dataset generalise to covariate groups, e.g. participant age. To model the generalisability of these performance measures, we compared the seropositivity classification of our study dataset using our in-house antibody assay, with the predicted seropositivity classification from hypothetical assays with a quoted sensitivity and specificity. Our”* These sentences are a little confusing. Firstly, what does ‘estimates’ in ‘estimates derived from our assay validation dataset generalise to covariate groups, e.g. participant age’ refer to? Is it referring to sensitivity and specificity? Secondly, “we compared the seropositivity classification of our study dataset using our in-house antibody assay” sounds like you compared the classification using the antibody assay. Thirdly, in “with the predicted seropositivity classification from hypothetical assays with a quoted sensitivity and specificity”, what does ‘quoted’ mean? Does it mean ‘assumed sensitivity and specificity since it is a hypothetical assay?

**David Hodgson: In answer to the reviewer’s first question; yes, “estimates” in this sentence refers to sensitivity and specificity. For the reviewer’s second point, we have clarified the sentence to ensure that this confusion does not arise.  Finally, for the reviewer's third point, yes, “quoted” in this sentence can be read as “assumed”. With these comments in mind, we have changed the text accordingly:**

**“In our heterogeneous sensitivity and specificity model, we explored how estimates for the sensitivity and specificity derived from our assay validation dataset generalise to covariate groups, e.g. participant age. To model the generalisability of these performance measures, we compared the seropositivity classification of our study dataset according to our in-house antibody assay, with the predicted seropositivity classification from hypothetical assays with an assumed sensitivity and specificity.”**

 8. Please introduce A450 when it was first mentioned in the manuscript.

**Hailey Hornsby: Added an introduction to A450 to Serology Assay Development: “Thresholds based on the absorbance value at 450nm (A 450) for defining reactivity to spike ( A 450 0·1750) or NCP ( A 450 0·1905) were set to optimise the sensitivity of each assay.**”

 9.In *“to model how reliably quoted performance measures generalise.”*, what does ‘quoted’ refer to? **David Hodgson: “Quoted” refers to the sensitivity and specificity given by the manufacturers of the assays. To make this clearer we have changed the text:**

**“to model how reliably the sensitivity and specificity given by the manufacturers of the assays  generalise to specific subpopulations.”**

 10. *“Using the assay validation dataset, we estimated the A450 cut-off value for a range of chosen sensitivity values, and then used this A450 cut-off to classify seropositivity in the study dataset.”* How did you choose a single A450 value from a range of sensitivity values?

**David Hodgson: There is an A450 value for each sensitivity value considered. To make this as clear as possible in the sentence we have changed the sentence accordingly:**

**“Using the assay validation dataset, we estimated an A450 cut-off value for every chosen   sensitivity value, and then used this A450 cut-off to classify seropositivity in the study dataset.”**

 11. “We then estimated the implied sensitivity on the HERO dataset by comparing seropositivity classification based on the estimated A450 cut-off value, with the seropositivity classification from our in-house assay (which for ease of comparison, we assume represents the maximum possible sensitivity and specificity (i.e. 100%) in this model).” What does ‘implied’ mean here?

**David Hodgson: “Implied sensitivity” is defined by the description following the sentence. To make this clearer we have rearranged the sentence accordingly:**

**“By comparing seropositivity classification based on the estimated A450 cut-off value, with the seropositivity classification from our in-house assay (which for ease of comparison, we assume represents the maximum possible sensitivity and specificity (i.e. 100%) in this model), we estimate an “implied” sensitivity on the HERO dataset which would arise if the commercial assay alone had been used to detect seropositivity.”**

 12. Please cite R and cmdstanr.

**David Hodgson:  Apologies for this oversight, these are now referenced in the updated manuscript.**

 Results:

 1. *“Rapid waning of IgA responses following SARS-CoV-2 infection complicated defining positive and negative samples based on the convalescent sera we used for assay validation.” *Why is this so?

**Hailey Hornsby: This sentence has been expanded and rewritten in manuscript to clarify:**

**“ Compared with IgG, we saw more rapid waning of the IgA responses following SARS-CoV-2 infection, a s well as higher levels of cross-reactivity in pre-pandemic samples. These factors complicated defining seropositivity based on an A450 threshold, as there was no clear separation between titres in these two groups. We therefore opted to use our spike and NCP IgA ELISA solely to compare IgA titres of individuals classified as seropositive by our IgG assay.”**

 2. *“1174 attended for a second visit (V2) between 15 June and 10 July 2020” *‘for’ is unnecessary in this sentence.

**Hayley Colton: Amended in manuscript:**

**“1174 attended a second visit (V2) between 15 June and 10 July 2020”**

 3.Figure 1: Why do the black stars not overlap with the unadjusted mean estimates?

**David Hodgson: As explained in the caption, the black stars are point estimates from the raw data (no regression was performed), calculated by as the mean value for that specific subgroup. The remaining estimates on the Figure are from a hierarchical regression analysis. We remind the reviewer that here “unadjusted” means relative to the primary exposure (job, zone, wards), and that regularisation from the hierarchical model means that estimates from this model are likely to be different from the raw point estimates. E.g. The oldest (60+ years) age group had a much smaller sample size compared to the other age groups, therefore it is more susceptible to regularisation in a regression analysis.**

 4. Why were zones, job and wards not used in the same model? Was their model selection done?

**David Hodgson: There were two issues with using more than one of the primary response variables (zones, jobs, and wards) in the model. First we found multicollinearity between these exposures, making parameter inference difficult to interpret when more than one primary exposure was included in the regression analysis model. Second, the number of categories of these age groups gave rise to data sparsity issues (many groups have 0 entries) meaning again, results were unreliable. These reasons are explained in the Extended Data (reference 21 in the manuscript).**

 5. Figure 1: The raw data showed that a higher proportion of HCWs who were >60 years old were seropositive compared to the other age groups. However, there’s no longer this association after adjustment. What was/were the confounder(s)?

**David Hodgson:  This is due to regularisation of the hierarchical regression model. The oldest (60+ years) age group had a much smaller sample size compared to the other age groups, therefore it is more susceptible to regularisation in a regression analysis. In other words, the magnitude of the high seroprevalence observed in the older age group (with such a small sample size) is unlikely to be a true reflection of the seroprevalence in that age groups, given the consistency in seroprevalence across the other age groups with a large sample size.**

 6. In the antibody kinetics model, neither zones, job and wards were used as an independent variable. As mentioned in the earlier point, would this finding of age and higher titres modulate/disappear if zones, job and/or wards are included in this model as well?

**David Hodgson: As explained in the response to the reviewers main comment above, the similarity between the unadjusted and adjusted seroprevalence estimates suggest that neither zone, jobs or wards were strong confounders on the age-dependent prevalence, therefore they are dropped in the subsequent modes for parsimony.**

 7. Figure 3: Please specify the meaning of ‘control’ in the x-axis.

**David Hodgson:** **Here the control means the “Sensitivity of assay validation dataset.” This change has been made to Figure 3.**

 Discussion:

 1. Were the infection prevention and control measures similar in the ED and AMU? Could the higher prevalence in AMU be due to transmissions from asymptomatic/ mildly symptomatic patients who were not triaged to be isolated by the ED?

**Hayley Colton: Local infection prevention and control measures were trust wide rather than department led, so it would be reasonable to presume they would be similar in both areas. Trust guidance was changed so HCWs should wear surgical masks, aprons and gloves for all patient contact from 08 April 2021, so it could be possible that transmissions in this manner could have been a factor prior to 08 April 2021. If the patient had been admitted to AMU via ED, they would have exposed HCWs in both the ED and AMU, however in the AMU the exposure time to HCWs is likely to be longer, plus patients could have come via GP and therefore not exposed ED HCWs. This has been added to the manuscript:**

**“ Despite universal PPE and IPC guidelines across STH, our data show that HCWs working in AMUs are at significantly higher risk of infection with SARS-CoV-2, with seropositivity rates above that of other wards, consistent with other UK studies. [...] Although some patient factors may increase HCW risk of infection on AMU compared to ED (cohort bays, longer stays of asymptomatic/paucisymptomatic cases prior to universal PPE use, more fomites e.g. bedside tables, chairs), more frequent interactions and therefore transmission between HCWs may also play a significant additional role”**

 2. *“Previous studies clearly demonstrate that patients with more severe COVID-19 have higher antibody titres”* However this was not found in this study?

**Hayley Colton: Our data looking at HCW antibody responses didn’t include information about severity of symptomatic COVID-19 infection in our HCWs, so we cannot say that we found, or did not find this in our study. However, we have added Figure S1b concerning our assay validation to our Extended Data, and referenced it in the manuscript.**

 3. *“Reassuringly, our seroprevalence rates are similar to those seen in other UK based seroprevalence studies” *Citation 5 also enrolled volunteer participants (publicised study using social media), and citation 21 is the HERO extended data?

**Hayley Colton/Paul Collini: The citations on this sentence are 5 and 32 which are referring to other UK seroprevalence studies on healthcare workers. We were trying to convey that our seroprevalence rates are similar to other UK HCWs, however I appreciate that the recruitment methods are similar to our study, which probably negates the point. I have rephrased as following:**

**“We note the limitations of our study, which include a potential for selection bias due to participants self-enrolling for convenience, rather than using systematic sampling. While we cannot measure the extent of this effect on the measured seroprevalence, we think any volunteer bias would have been equal across all categories we compared, and so not altering the validity of these comparisons.”**

 4. It will be informative to have a more detailed discussion on if the finding of age affecting serology titres were reported in other studies

**Hayley Colton: Thank you - we have added further detail to the age section in the discussion:**

**“ Our data complements the existing literature, which shows antibody titres against SARS-CoV-2 and other coronaviruses are higher in older individuals, which could be due to a higher risk of exposure to the virus, greater antigenic load, or boosting of antibodies from cumulative seasonal coronavirus infections throughout their lifetime15-19.”**
